# The Role of Medicaid Dental Benefits in Access to Preventive Dental Care and Unmet Dental Needs During Pregnancy

**DOI:** 10.21203/rs.3.rs-8340673/v1

**Published:** 2025-12-18

**Authors:** Cilia E. Zayas, Sarah H. Gordon, Hawazin W. Elani, Astha Singhal

**Affiliations:** University of Florida; Boston University; Harvard University, The Harvard T.H. Chan School of Public Health; University of Florida

**Keywords:** Medicaid, dental access, pregnancy, preventive care, maternal health

## Abstract

**Background:**

Maintaining oral health during pregnancy is important for maternal and child health, yet many lowincome individuals face barriers to dental care. Medicaid dental benefits vary by state and may influence access to preventive services and unmet dental needs during pregnancy. This study examined whether state Medicaid dental benefits for pregnant women were associated with preventive dental care use and unmet dental needs, overall and by race and ethnicity.

**Methods:**

We analyzed data from the Pregnancy Risk Assessment Monitoring System for 45 states from 2012 to 2021 using a pooled cross-sectional design. The sample included Medicaid-enrolled pregnant women. Outcomes were receipt of a dental cleaning and reporting unmet dental needs during pregnancy. State Medicaid dental benefit status was the primary exposure. Descriptive and bivariate analyses summarized sample characteristics and outcome prevalence. Multivariable regression models estimated adjusted differences in outcomes by benefit status. Subgroup analyses were conducted for Hispanic, non-Hispanic White, non-Hispanic Black, and non-Hispanic Other groups.

**Results:**

Among Medicaid-enrolled pregnant women, 34.1% reported a dental cleaning and 10.8% reported unmet dental needs. Living in a state with Medicaid dental benefits was associated with a 6.7 percentage point higher likelihood of receiving a dental cleaning (95% CI: 4.0, 9.5) and a 4.0 percentage point lower likelihood of reporting unmet dental needs (95% CI: −6.4, − 1.7). The association with dental cleaning was strongest among non-Hispanic Black women (9.1 percentage points; 95% CI: 2.6, 15.6), followed by Hispanic women (8.3; 95% CI: 1.6, 15.1) and non-Hispanic White women (5.7; 95% CI: 2.0, 9.4). A significant association with reduced unmet needs was observed only among non-Hispanic White women.

**Conclusions:**

State Medicaid dental benefits were associated with greater use of preventive dental care and fewer unmet dental needs during pregnancy. Expanding dental coverage for pregnant Medicaid beneficiaries may improve access to oral health care and help reduce disparities.

## Background

Poor oral health during pregnancy can lead to complications such as pre-eclampsia (1, 2), preterm birth and low birth weight.(3, 4). Pregnancy may induce a hyperinflammatory state that exacerbates periodontal disease (5). Regular dental cleanings help remove bacterial plaque and reduce inflammation, potentially lowering the risk of periodontal disease (6). Despite these implications, only 46% of pregnant women reported receiving a dental cleaning during pregnancy in 2016–2017 (7).

Cost remains a significant barrier to accessing dental care, particularly for low-income populations (8, 9). Medicaid finances approximately 41% of all births in the United States, providing essential coverage to low-income, rural, and minority women who face elevated risks of maternal morbidity and mortality (10). Yet, only 28% of Medicaid-enrolled pregnant women report receiving a dental cleaning during pregnancy (10). This gap may reflect variation in state Medicaid policies, as adult dental coverage—including for pregnant women—is an optional benefit under federal Medicaid guidelines and varies widely by state (11, 12).

Previous studies suggest that Medicaid dental benefits may improve access to dental care and reduce emergency department visits for dental problems among low-income populations (13–15). Some research also indicates that Medicaid dental coverage may be linked to higher rates of dental cleanings during pregnancy (13, 16). However, these studies have not examined whether these associations differ across racial and ethnic groups—an important gap given persistent inequities in oral health access.

Racial and ethnic disparities in dental care access are well documented, particularly among low-income and publicly insured populations (17–21). Few studies have focused on disparities during pregnancy, a period when oral health is closely linked to maternal and infant outcomes. A recent Pregnancy Risk Assessment Monitoring System (PRAMS) analysis found that fewer than half of pregnant women received dental cleanings, with significantly lower rates among non-White women (17). The lowest predicted rates were among Black (43.2%) and Asian and Pacific Islander (API) (30.6%) women (17). Among Medicaid enrollees, API women had the lowest predicted rate of dental cleanings during pregnancy (25.4%) compared to privately insured White women (57.4%) (17). These disparities highlight the need to examine whether Medicaid dental benefits are equitably associated with improved access across racial and ethnic groups.

Using PRAMS data from 45 states over a nine-year period (2012–2021) (22), this study examined the association between state Medicaid dental benefits and access to dental care during pregnancy among adult Medicaid-enrolled women. Access was assessed using two outcomes: receipt of a dental cleaning and reporting an unmet dental need during pregnancy. Unmet need was defined as respondents who indicated they “needed to see a dentist about a problem” but “did not see a dentist about a problem.” We also evaluated whether these associations varied across racial and ethnic groups to explore potential inequities in access to dental care.

## Methods

### Study Design and Data Source

This study used a pooled cross-sectional design to analyze data from the Pregnancy Risk Assessment Monitoring System collected between 2012 and 2021. PRAMS is a state-based, population-level surveillance system that collects information on maternal attitudes and experiences before, during, and shortly after pregnancy. (22). Data are collected through mailed surveys administered to a representative sample of women who recently gave birth, with typical response rates around 70 percent. A list of states included in the analysis is provided in Appendix 1. Because PRAMS data are de-identified and publicly available, the University of Florida Institutional Review Board determined this study to be exempt from review.

## Study Population

The study population included PRAMS respondents aged 21 years and older who had a live birth during the study period and reported that Medicaid covered their prenatal care. Individuals under age 21 were excluded due to differences in Medicaid pregnancy-related policies. The initial dataset included 387,149 respondents who had a live birth during the study period. Appendix 2 outlines the sample selection process and the distribution of study outcomes.

## Measures

The primary outcomes were two binary indicators of dental care access during pregnancy. The first outcome measured preventive dental care, defined as whether the respondent reported having their teeth cleaned by a dentist or dental hygienist during pregnancy. The second outcome captured unmet dental need, defined as whether the respondent reported needing to see a dentist for a problem but did not receive care.

The main exposure variable was the availability of state Medicaid dental benefits for pregnant beneficiaries. Information on state-level Medicaid dental benefits for adult pregnant beneficiaries were compiled from multiple sources, including an Excel spreadsheet shared via personal communication with M. Vujicic and C. Yarborough (July 19, 2019), which detailed state-level adult Medicaid dental benefits for pregnant enrollees. Additional sources included the Medicaid and CHIP Payment and Access Commission (23), and Medicaid State Plan Amendments (24). Following prior literature, states were categorized into two groups: those offering dental benefits (including limited or extensive coverage) and those not offering benefits (including emergency-only or no coverage). (25, 26). This binary classification was used because preventive services such as dental cleaning are typically covered under both limited and extensive plans but not under emergency-only or no coverage.

Covariates included a range of sociodemographic and health-related characteristics. These included age group, race and ethnicity (Hispanic, non-Hispanic White, non-Hispanic Black, and non-Hispanic Other), pregnancy intention (intended versus unintended), education level, marital status, household income, body mass index (BMI) category, pre-pregnancy chronic conditions (including diabetes, hypertension, and depression), number of previous live births, and the trimester in which prenatal care was initiated. The non-Hispanic Other category included PRAMS maternal race groups such as Other Asian, American Indian, Chinese, Japanese, Filipino, Other Non-White, Alaska Native, and individuals of mixed race.

### Statistical Analysis

Descriptive statistics were used to summarize sample characteristics and outcome distributions. Bivariate associations between Medicaid dental benefits, race and ethnicity, and dental care outcomes were assessed using Chi-square tests. Multivariable regression models were used to estimate the association between offering Medicaid dental benefits (limited /extensive) versus not offering dental benefits (emergency / no benefits) and the likelihood of receiving a dental cleaning or experiencing an unmet dental need during pregnancy, adjusting for covariates. All models included state and year fixed effects to account for unobserved heterogeneity. Stratified analyses were conducted to examine whether associations between Medicaid dental benefits and dental care outcomes varied among different racial and ethnic groups. Separate multivariable models were estimated for Hispanic, non-Hispanic White, nonHispanic Black, and non-Hispanic Other women. All analyses accounted for PRAMS’ complex sampling design and were conducted using SAS version 9.4 and Stata version 17.0. Statistical significance was defined as p < 0.05, and 95% confidence intervals were reported.

## Results

Among pregnant women aged 21 years and older who had a live birth during the study period (2012–2021), 47.7% reported receiving a dental cleaning during pregnancy, while 6.3% reported experiencing an unmet dental need. Among Medicaid-enrolled pregnant women, 34.1% reported receiving a dental cleaning, and 10.8% reported an unmet dental need. Table 1 presents sample characteristics and bivariate associations between key variables and study outcomes. Both Medicaid adult dental benefits (yes/no) and race and ethnicity were significantly associated with receipt of dental cleaning and unmet dental needs during pregnancy (p < 0.001).

### Multivariable Analysis

Multivariable regression results are summarized in Table 2. Residing in a state that offered pregnancy-related adult Medicaid dental benefits was associated with a 6.7 percentage point higher likelihood of receiving a dental cleaning (95% CI: 4.0, 9.5) and a 4.0 percentage point lower likelihood of reporting an unmet dental need during pregnancy (95% CI: −6.4, −1.7). After adjusting for covariates, Hispanic women were 6.3 percentage points more likely to receive a dental cleaning (95% CI: 4.7, 7.7) and 3.8 percentage points less likely to report an unmet dental need during pregnancy (95% CI: −4.9, −2.7) compared to non-Hispanic White women. Similarly, non-Hispanic Black women were 4.1 percentage points more likely to receive a dental cleaning (95% CI: 2.8, 5.4) and 2.2 percentage points less likely to report an unmet dental need during pregnancy (95% CI: −3.2, −1.2).

Sociodemographic factors were also associated with dental care outcomes. Women with a college degree or higher were 8.4 percentage points more likely to receive a dental cleaning (95% CI: 6.5, 10.2) and 3.1 percentage points less likely to report an unmet dental need during pregnancy (95% CI: −4.5, −1.7) compared to those with a high school education or less. Higher household income was associated with greater likelihood of receiving a dental cleaning and lower likelihood of reporting unmet dental needs during pregnancy. For example, women with household incomes above $60,000 were 7.7 percentage points more likely to report a dental cleaning (95% CI: 5.1, 10.2) and 3.9 percentage points less likely to report an unmet dental need (95% CI: −5.8, −2.0) compared to those with incomes below $20,000.

Pregnancy intention and timing of prenatal care initiation were also significantly associated with outcomes. Women who intended to become pregnant were 2.6 percentage points more likely to receive a dental cleaning (95% CI: 1.6, 3.7) and 2.2 percentage points less likely to report an unmet dental need during pregnancy (95% CI: −3.0, −1.4), compared to those with unintended pregnancies. Initiating prenatal care in the second or third trimester was associated with a 6.8 percentage point lower likelihood of receiving a dental cleaning (95% CI: −8.0, −5.5) and a 3.5 percentage point higher likelihood of reporting an unmet dental need during pregnancy (95% CI: 2.4, 4.5), compared to initiating care in the first trimester. Pre-pregnancy hypertension was associated with a higher likelihood of receiving a dental cleaning (2.5 percentage points; 95% CI: 0.4, 4.6), while pre-pregnancy depression was associated with a higher likelihood of reporting an unmet dental need (4.4 percentage points; 95% CI: 3.3, 5.6) during pregnancy.

## Stratified Analysis by Race and Ethnicity

To examine whether the association between pregnancy-related state Medicaid adult dental benefits and dental care access during pregnancy differed by race and ethnicity, we conducted separate multivariable models for each racial and ethnic group. These subgroup analyses assessed the relationship between Medicaid dental benefits for pregnant beneficiaries and two outcomes: receipt of a dental cleaning and reporting an unmet dental need during pregnancy. Results for Hispanic, non-Hispanic Black, non-Hispanic White, and non-Hispanic Other women are summarized in Table 3, with full model estimates provided in Appendix 3 and Appendix 4.

Among Hispanic, non-Hispanic Black, and non-Hispanic White women, residing in a state that offered Medicaid dental benefits was associated with a higher likelihood of reporting a dental cleaning during pregnancy. The association was largest among non-Hispanic Black women (9.1 percentage points; 95% CI: 2.6, 15.6), followed by Hispanic women (8.3 percentage points; 95% CI: 1.6, 15.1) and non-Hispanic White women (5.7 percentage points; 95% CI: 2.0, 9.4). Among non-Hispanic Other women, the association was not statistically significant (4.3 percentage points; 95% CI: −2.3, 10.9).

For unmet dental needs, Medicaid dental benefits were associated with a lower likelihood of reporting unmet needs during pregnancy only among non-Hispanic White women (−4.5 percentage points; 95% CI: −7.8, −1.1). The associations were not statistically significant for Hispanic (−4.6 percentage points; 95% CI: −10.6, 1.3), non-Hispanic Black (−3.2 percentage points; 95% CI: −8.1, 1.7), or non-Hispanic Other individuals (−2.1 percentage points; 95% CI: −11.3, 7.0).

## Discussion

This study examined the association between pregnancy-related adult Medicaid dental benefits and access to dental care during pregnancy among Medicaid-enrolled women, with a focus on racial and ethnic differences. Findings indicate that adult Medicaid dental benefits were associated with a higher likelihood of receiving a dental cleaning and a lower likelihood of reporting an unmet dental need during pregnancy. These associations varied by race and ethnicity. After adjusting for confounders, Hispanic and non-Hispanic Black women were more likely to report receiving a dental cleaning and less likely to report unmet dental needs during pregnancy compared to non-Hispanic White women. The largest adjusted difference in dental cleaning was observed among non-Hispanic Black women, followed by Hispanic and non-Hispanic White women. However, a significant association between Medicaid dental benefits and reduced unmet dental needs was observed only among non-Hispanic White women.

These findings highlight the variability in how pregnancy-related adult Medicaid dental benefits are associated with dental care outcomes across racial and ethnic groups. While the results suggest that such benefits may help reduce disparities in access to preventive dental care, similar patterns were not observed for unmet dental needs, which may reflect more complex barriers to accessing dental services among minoritized populations.

The results align with prior studies demonstrating the positive association between Medicaid dental benefits and dental care utilization among low-income adults. Among pregnant individuals, limited or no Medicaid dental coverage has been associated with a lower likelihood of receiving dental cleanings during pregnancy (13, 16). Additionally, having dental insurance alongside Medicaid medical coverage has been shown to increase the likelihood of receiving dental cleaning during pregnancy (27). These findings are particularly relevant for low-income racial and ethnic minority groups, who often face greater barriers to accessing dental care.

Previous research has also shown that Medicaid dental benefits are associated with increased dental care utilization among non-Hispanic Black and Hispanic adults (28). Comprehensive dental coverage through Medicaid has been especially effective in improving access among these groups, potentially contributing to reductions in disparities and advancing health equity (29). However, access to dental care is shaped by a range of factors beyond insurance coverage. Barriers such as perceived discrimination, lack of awareness about available services, and cultural or language differences may limit the effectiveness of coverage expansions (30–33). Cultural and language barriers also play a crucial role, as they can lead to misunderstandings about dental procedures and reluctance to seek care (34, 35). Provider availability, which may be influenced by reimbursement rates and geographic distribution, also plays a critical role (36). Additionally, concerns about the safety of dental procedures during pregnancy may deter individuals from seeking care (37). Addressing these barriers requires a comprehensive approach that includes enhancing Medicaid dental benefits, improving provider availability, and increasing awareness about the safety and importance of dental care during pregnancy (37).

Our study also found that the association between adult Medicaid dental benefits and reduced unmet dental needs during pregnancy was only significant among non-Hispanic White women. Several factors could potentially explain this finding. Non-Hispanic White women may have higher levels of health literacy and awareness about the availability and importance of dental services during pregnancy (38). Additionally, non-Hispanic White women may experience fewer barriers related to perceived discrimination and trust in healthcare providers (39). Cultural and language barriers, which can affect how racial and ethnic minority groups perceive and utilize dental care, may be less prevalent among non-Hispanic White women (40). Finally, concerns about the safety of dental procedures during pregnancy and or lower oral health literacy may be more common among racial and ethnic minority groups, leading to lower utilization of dental services despite having coverage (41).

Future research should explore the long-term effects of Medicaid dental benefits and assess whether expanded benefits lead to sustained improvements in maternal and infant health outcomes. Additionally, future studies should investigate implementation barriers in states with dental coverage, such as provider availability and administrative hurdles, to examine why certain populations, such as non-Hispanic Black women, do not experience reductions in unmet dental needs despite having dental coverage. Although this study did not find a significant relationship between offering Medicaid dental benefits and reporting unmet dental needs during pregnancy among Hispanic and non-Hispanic Black women, other literature suggests that perceived discrimination, lack of awareness, dental fear and anxiety, and feelings of self-consciousness or embarrassment due to their oral health may contribute to higher unmet dental needs among non-Hispanic Black women (31, 42–44). Perceived discrimination and low oral health literacy also appear to contribute to racial-ethnic disparities in dental utilization among Hispanics (32, 45). Understanding these barriers could inform more effective interventions to improve dental care access for Hispanic and non-Hispanic Black women. Further investigation into disparities related to unmet dental needs during pregnancy is warranted to address unequal access to dental care.

This study has several strengths, including the use of a large, nationally representative dataset and a comprehensive analysis of pregnancy-related adult Medicaid dental benefits across multiple states and years. Moreover, the study evaluates both preventive dental care (dental cleaning) and unmet dental needs during pregnancy as perceived by the respondents. However, the cross-sectional design limits causal inference, and reliance on self-reported data may introduce reporting bias. Despite these limitations, our findings offer valuable insights into the association between adult Medicaid dental benefits and dental care access and unmet needs among low-income pregnant women.

## Conclusion

Pregnancy-related adult Medicaid dental benefits were associated with differences in reported access to dental care and unmet dental needs among pregnant women, with variation across racial and ethnic groups. While such benefits may support more equitable access to preventive dental care, our findings did not show a significant association between Medicaid dental benefits and reduced unmet dental needs among Hispanic and non-Hispanic Black women, suggesting that additional barriers may contribute to persistent disparities. Expanding these benefits remains an important step, but further efforts are likely needed to address structural, cultural, and interpersonal barriers to care. Future research should explore the long-term effects of these benefits and identify strategies to ensure that all populations can equitably benefit from expanded coverage.

## Supplementary Material

Supplementary Files

This is a list of supplementary files associated with this preprint. Click to download.

- Appendix1ListofStatesIncludedintheAnalysis.docx
- Appendix2SampleSelectionandOutcomeDistribution.docx
- Appendix3SubgroupAnalysisDentalCleaning2025MAY22.docx
- Appendix4SubgroupAnalysisUnmetDentalNeeds2025MAY22.docx

## Figures and Tables

**Table 1:** Bivariate Analysis of Dental Cleaning and Unmet Dental Needs During Pregnancy Among MedicaidEnrolled Women, 2012–2021

	Received Dental Cleaning During Pregnancy			Reported Unmet Dental Need During Pregnancy		
	Unweighted n = 32,952			Unweighted n = 8,020		
	Weighted % = 34.1%			Weighted % = 10.8		
Factors	Unweighted (n)	Weighted (%)	p-value	Unweighted (n)	Weighted (%)	p-value
**Medicaid dental benefits**			**< 0.001**			**< 0.001**
Offered dental benefits	27,709	36.0%		5,281	9.5%	
Did not offer dental benefits	5,243	24.8%		2,739	17.9%	
**Age Categories**			**0.006**			0.300
21–24	8,192	33.0%		1,921	10.2%	
25–29	11,377	34.0%		2,846	11.2%	
30–34	8,114	34.5%		2,039	10.9%	
35+	5,269	35.9%		1,214	89.4%	
**Race-Ethnicity**			**< 0.001**			**< 0.001**
Hispanic	5,937	39.4%		814	6.8%	
NH Black	9,959	33.7%		2,289	10.7%	
NH Other	5,689	32.8%		1,113	10.1%	
NH White	11,367	32.9%		3,804	12.2%	
**Pregnancy Intention**			**< 0.001**			**< 0.001**
Intended	11,550	36.8%		2,092	8.4%	
Unintended	21,402	32.8%		5,928	11.9%	
**Marital Status**			**< 0.001**			**< 0.001**
Married	11,462	36.3%		2,337	9.3%	
Unmarried	21,490	33.1%		5,683	11.5%	
**Maternal Education**			**< 0.001**			**< 0.001**
High School Graduate	16,156	31.7%		4,543	11.5%	
Some College	12,839	35.3%		2,978	10.9%	
College Degree or Higher	3,957	42.7%		499	6.4%	
**Household Income**			**< 0.001**			**< 0.001**
Below $20,000	17,044	32.1%		5,141	12.5%	
$20,000 to $40,000	10,624	35.2%		2,144	9.5%	
$40,001 to $60,000	3,512	31.2%		537	7.5%	
Above $60,000	1,772	43.0%		198	6.7%	
**Diabetes Before Pregnancy**			0.615			0.468
Yes	1,282	34.8%		335	11.5%	
No	31,670	34.1%		7,685	10.7%	
**High Blood Pressure Before Pregnancy**			0.344			**0.018**
Yes	2,512	35.0%		794	12.4%	
No	30,440	34.1%		7,226	10.6%	
**Depression Before Pregnancy**			**< 0.001**			**< 0.001**
Yes	6,050	32.0%		2,327	15.4%	
No	26,902	34.6%		5,693	9.7%	
**BMI Category**			**< 0.001**			**0.007**
BMI < 18.5 (Underweight)	1,252	31.3%		427	13.6%	
18.5 < = BMI < = 24.9 (Healthy weight)	12,737	35.4%		3,097	10.9%	
25.0 < = BMI < 30.0 (Overweight)	8,544	34.8%		1,855	10.0%	
BMI > 30.0 (Obese)	10,419	32.5%		2,641	10.8%	
**Number of Previous Live Births**			0.438			**< 0.001**
0	9,508	33.9%		2,115	9.6%	
1	10,006	33.9%		2,353	10.4%	
2 or more	13,438	34.5%		3,552	12.0%	
**First Prenatal Care Visit**			**< 0.001**			**< 0.001**
1st Trimester (weeks 1–12)	28,150	35.4%		6,038	10.0%	
2nd or 3rd Trimester (weeks > = 13)	4,802	28.1%		1,982	14.6%	

**Note**: The non-Hispanic Other category includes the following PRAMS maternal race groups: Other Asian, American Indian, Chinese, Japanese, Filipino, Other Non-White, Alaska Native, and individuals of mixed race.

**Table 2:** Multivariable Analysis of Dental Cleaning and Unmet Dental Needs During Pregnancy Among MedicaidEnrolled Women, 2012–2021

	Received dental cleaning during pregnancy			Reported unmet dental need during pregnancy		
Factor	Marginal effects in percentage point difference	95% CI	p-Value	Marginal effects in percentage point difference	95% CI	p-Value
**Medicaid Dental Benefits**						
Offered dental benefits	6.7	[4.0, 9.5]	**< 0.001**	−4.0	[−6.4, −1.7]	**0.001**
Did not offer dental benefits (Ref)	-	-	-	-	-	-
**Age Category**						
25–29	−0.9	[−2.3, 0.4]	0.173	1.7	[0.7, 2.7]	**0.001**
30–34	−1.6	[−3.1, −0.1]	**0.036**	2.1	[0.9, 3.2]	**< 0.001**
35+	−1.2	[−3.0, 0.5]	0.176	2.2	[0.7, 3.6]	**0.003**
21–24 (Ref)	-	-	-	-	-	-
**Race-Ethnicity**						
Hispanic	6.3	[4.8, 7.8]	**< 0.001**	−3.8	[−4.9, −2.7]	**< 0.001**
NH Black	4.1	[2.8, 5.4]	**< 0.001**	−2.2	[−3.2, −1.2]	**< 0.001**
NH Other	−1.2	[−2.8, 0.4]	0.129	−0.3	[−1.9, 1.2]	0.663
NH White (Ref)	-	-	-	-	-	-
**Pregnancy Intention**						
Intended	2.6	[1.6, 3.7]	**< 0.001**	−2.2	[−3.0, −1.4]	**< 0.001**
Unintended (Ref)	-	-	-	-	-	-
**Marital Status**						
Married	0.9	[−0.2, 2.0]	0.100	−1.1	[−2.0, −0.3]	**0.010**
Unmarried (Ref)	-	-	-	-		-
**Maternal Education**						
Some College	2.5	[1.5, 3.6]	**< 0.001**	0.2	[−0.6, 1.0]	0.643
College Degree or Higher	8.4	[6.5, 10.2]	**< 0.001**	−3.1	[−4.5, −1.7]	**< 0.001**
High school graduate or less (Ref)	-	-	-	-		-
**Household Income**						
$20,000-$40,000	1.9	[0.8, 3.1]	**0.001**	−1.8	[−2.7, −0.9]	**< 0.001**
$40,001-$60,000	2.9	[1.2, 4.7]	**0.001**	−3.6	[−4.8, −2.3]	**< 0.001**
More than $60,000	7.7	[5.1, 10.2]	**< 0.001**	−3.9	[−5.8, −2.0]	**< 0.001**
Below $20,000 (Ref)	-	-	-	-		-
**Diabetes Before Pregnancy**						
Yes	−0.2	[−3.0, 2.5]	0.885	−0.4	[−2.5, 1.6]	0.695
No (Ref)	-	-	-	-		-
**High Blood Pressure Before Pregnancy**						
Yes	2.5	[0.4, 4.6]	**0.017**	−0.1	[−1.6, 1.3]	0.848
No (Ref)	-	-	-	-	-	-
**Depression Before Pregnancy**						
Yes	−0.6	[−1.9, 0.7]	0.380	4.4	[3.3, 5.6]	**< 0.001**
No (Ref)	-	-	-	-	-	-
**BMI Category**						
Underweight	−2.9	[−5.4, – −0.4]	**0.022**	1.7	[−0.3, 3.8]	0.100
Overweight	−0.9	[−2.2, 0.3]	0.147	−0.9	[−1.9, 0.1]	0.084
Obese	−2.4	[−3.6, −1.3]	**< 0.001**	−0.7	[−1.6, 0.2]	0.152
Healthy Weight (Ref)	-	-	-	-	-	-
**Number of Previous Live Births**						
1	0.3	[−1.0, 1.5]	0.658	0.7	[−0.3, 1.7]	0.188
2+	2.2	[0.9, 3.5]	**0.001**	1.5	[0.4, 2.5]	**0.006**
0 (Ref)	-	-	-	-	-	-
**First Prenatal Care Visit**						
2nd or 3rd Trimester	−6.8	[−8.0, −5.5]	**< 0.001**	3.5	[2.4, 4.5]	**< 0.001**
First Trimester (Ref)	-	-	-	-	-	-

**Note** (1): Marginal effects from a multivariable logistic regression were used to assess the association between Medicaid dental benefits and the probability of receiving a dental cleaning. Results are expressed as percentage point differences in the predicted probability (risk) for ease of interpretation.

**Note** (2): Due to the low prevalence of unmet dental needs, a multivariable Poisson regression was used to model this outcome. Marginal effects were calculated and are expressed as percentage point differences in the predicted probability of unmet dental needs for interpretability.

**Note** (3): States were categorized based on whether they offered dental benefits to Medicaid-enrolled pregnant women. “Offered dental benefits” includes states with limited or extensive dental coverage, while “Did not offer dental benefits” includes states with emergency-only or no dental coverage. This categorization was chosen because dental cleaning during pregnancy is covered under limited or extensive benefits but not under emergency-only or no benefits.

**Note** (4): The non-Hispanic Other category includes the following PRAMS maternal race groups: Other Asian, American Indian, Chinese, Japanese, Filipino, Other Non-White, Alaska Native, and individuals of mixed race.

**Note** (5): State and year fixed effects were included to control for unobserved heterogeneity, which refers to unmeasured factors at the state and year levels that could influence the outcome (e.g., dental cleaning or unmet dental needs) but are not directly captured by the model.

**Note** (6): Data from 45 states were analyzed for dental cleaning during pregnancy, excluding observations with missing values.

**Note** (7): For unmet dental needs during pregnancy, data from 42 states were included, excluding Florida, Kansas, and South Dakota due to the absence of data on this outcome.

**Table 3:** Racial and Ethnic Subgroup Analysis of Dental Cleaning and Unmet Dental Needs During Pregnancy by Medicaid Dental Benefit Status, 2012–2021

	Received Dental Cleaning During Pregnancy			Reported Unmet Dental Need During Pregnancy		
Race-Ethnicity	Marginal Effects in percentage point difference	95% CI	p-Value	Marginal Effects in percentage point difference	95% CI	p-Value
Hispanic	8.3	[1.6, 15.1]	**0.016**	−4.6	[−10.6, 1.3]	0.126
NH Black	9.1	[2.6, 15.6]	**0.006**	−3.2	[−8.1, 1.7]	0.197
NH White	5.7	[2.0, 9.4]	**0.003**	−4.5	[−7.8, −1.1]	**0.010**
NH Other	4.3	[−2.3, 10.9]	0.200	−2.1	[−11.3, 7.0]	0.649

**Note** (1): Marginal effects, derived from logistic and Poisson regression models, represent percentage point differences in the predicted probability (risk) of each outcome for individuals in states with versus without Medicaid dental benefits, within each racial and ethnic group. Logistic regression was used for dental cleaning; Poisson regression was used for unmet dental needs due to low outcome prevalence.

**Note** (2): The non-Hispanic Other category includes the following PRAMS maternal race groups: Other Asian, American Indian, Chinese, Japanese, Filipino, Other Non-White, Alaska Native, and individuals of mixed race.

**Note** (3): Covariates included, age categories, racial and ethnic groups, pregnancy intention, education level, marital status, household income, Body Mass Index categories, chronic health conditions before pregnancy, such as diabetes, high blood pressure, and depression, number of previous live birth(s) and trimester of first prenatal care visit.

**Note** (4): State and year fixed effects were included to control for unobserved heterogeneity, which refers to unmeasured factors at the state and year levels that could influence the outcome (e.g., dental cleaning or unmet dental needs) but are not directly captured by the model.

## Data Availability

All data is freely available to the public from the Centers for Disease Control and Prevention, Pregnancy Risk Assessment Monitoring System.
